# Lineage-Specific Expression Divergence in Grasses Is Associated with Male Reproduction, Host-Pathogen Defense, and Domestication

**DOI:** 10.1093/gbe/evy245

**Published:** 2018-11-06

**Authors:** Raquel Assis

**Affiliations:** Department of Biology, Pennsylvania State University, University Park

**Keywords:** lineage-specific evolution, expression evolution, domestication

## Abstract

Poaceae (grasses) is an agriculturally important and widely distributed family of plants with extraordinary phenotypic diversity, much of which was generated under recent lineage-specific evolution. Yet, little is known about the genes and functional modules involved in the lineage-specific divergence of grasses. Here, I address this question on a genome-wide scale by applying a novel branch-based statistic of lineage-specific expression divergence, LED, to RNA-seq data from nine tissues of the wild grass *Brachypodium distachyon* and its domesticated relatives *Oryza sativa japonica* (rice) and *Sorghum bicolor* (sorghum). I find that LED is generally smallest in *B. distachyon* and largest in *O. sativa japonica*, which underwent domestication earlier than *S. bicolor*, supporting the hypothesis that domestication may increase the rate of lineage-specific expression divergence in grasses. Moreover, in all three species, LED is positively correlated with protein-coding sequence divergence and tissue specificity, and negatively correlated with network connectivity. Further analysis reveals that genes with large LED are often primarily expressed in anther, implicating lineage-specific expression divergence in the evolution of male reproductive phenotypes. Gene ontology enrichment analysis also identifies an overrepresentation of terms related to male reproduction in the two domesticated grasses, as well as to those involved in host-pathogen defense in all three species. Last, examinations of genes with the largest LED reveal that their lineage-specific expression divergence may have contributed to antimicrobial functions in *B. distachyon*, to enhanced adaptation and yield during domestication in *O. sativa japonica*, and to defense against a widespread and devastating fungal pathogen in *S. bicolor*. Together, these findings suggest that lineage-specific expression divergence in grasses may increase under domestication and preferentially target rapidly evolving genes involved in male reproduction, host-pathogen defense, and the origin of domesticated phenotypes.

## Introduction

Grasses are flowering plants that comprise many economically important crops, including rice, wheat, and maize. Adaptations to diverse environments ranging from lush rainforests to cold deserts enabled ancient grasses to inhabit every continent of the globe, including Antarctica, providing food and energy for numerous organisms (Bouchenak-Khelladi et al. 2010). It may be for this reason that grasses were the first plants to be domesticated by humans ∼12,000 years ago, fueling the transition from hunting and gathering to agriculture (Glémin and Bataillon 2009). Under domestication, many grasses experienced accelerated phenotypic evolution, such that contemporary variants comprising a majority of the modern human diet often bear little resemblance to the ancestral species from which they arose (Glémin and Bataillon 2009). Due to this extraordinary diversity generated within a short evolutionary timeframe, grasses represent a unique system in which to examine lineage-specific phenotypic divergence among closely related species.

Widespread conservation of the genetic repertoire across the tree of life has led many to hypothesize that phenotypic divergence often occurs via mutations that affect the regulation of gene expression (King and Wilson 1975; Wray et al. 2003; Carroll 2005, 2008). In particular, perturbations in the level, location, or timing of expression can dramatically alter the function of a gene and, as a result, the phenotype of an organism (Carroll 2008; Liao and Weng 2015). Moreover, such changes are often strongly correlated with diverse genic properties, including protein-coding sequence divergence (Makova and Li 2003; Nuzhdin et al. 2004; Jordan et al. 2005; Lemos et al. 2005; Duret and Mouchiroud 2000; Pal et al. 2001; Herbeck et al. 2003; Rocha and Danchin 2004; Subramanian and Kumar 2004; Assis 2014; Assis and Kondrashov 2014; Sartor et al. 2006; Assis et al. 2012; Assis and Bachtrog 2013, 2015; Hunt et al. 2013; Gossmann et al. 2016; Hodgins et al. 2016; Mähler et al. 2017), expression breadth (Meiklejohn et al. 2003; Jordan et al. 2005; Duret and Mouchiroud 2000; Subramanian and Kumar 2004; Bhardwaj and Lu 2005; Assis and Bachtrog 2013; Assis 2014; Assis and Kondrashov 2014; Assis et al. 2012; Assis and Bachtrog 2013, 2015; Gossmann et al. 2016), and network connectivity (Lemos et al. 2005; Assis and Bachtrog 2013; Bhardwaj and Lu 2005; French and Pavlidis 2011; Assis and Kondrashov 2014; Mähler et al. 2017). Thus, how, where, and when a gene is expressed—its expression profile—is frequently considered an ideal proxy for its function (Nehrt et al. 2011; Assis and Bachtrog 2013, 2015; De Smet et al. 2017). Further, in contrast to alternative metrics of gene function, expression profiles are easily quantified, compared, and interpreted.

In a recent study, Davidson et al. (2012) generated RNA-seq data from nine tissues of three grass species: *Brachypodium distachyon*, *Oryza sativa japonica* (rice), and *Sorghum bicolor* (sorghum). *Brachypodiumdistachyon* and *O. sativa japonica* are sisters whose lineages diverged from each other ∼50 Ma, and from that of their close outgroup *S. bicolor* ∼60 Ma (Paterson et al. 2009; Reineke et al. 2011). Comparison of these grass transcriptomes revealed that most protein-coding genes are shared among the three species, but that orthologous genes often occupy distinct coexpression clusters (Davidson et al. 2012), supporting the hypothesis that mutations affecting gene expression played a central role in the phenotypic divergence of grasses. Yet an untapped utility of these RNA-seq data is that they enable the study of lineage-specific expression divergence, which can provide insight into phenotypic divergence that occurred along specific grass lineages. Thus, here I use these data to quantify lineage-specific expression divergence in grasses and explore its role in domestication, characterize its relationships with genic properties, and assess its functional targets.

## Results

### Quantification of Lineage-Specific Expression Divergence in Grasses

**Fig. 1.**
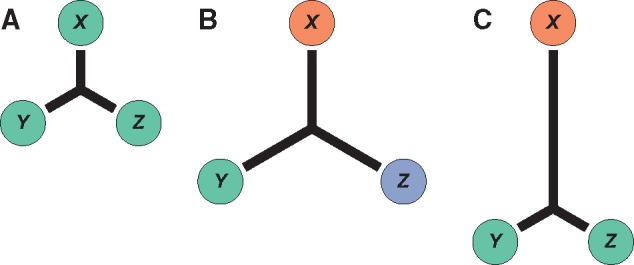
—A branch-based approach for quantifying lineage-specific expression divergence from gene expression profiles in three species. Depicted are unrooted trees of three orthologous genes in species *X*, *Y*, and *Z*, with their expression profiles illustrated as colored circles, and branch lengths representing their expression divergence from the internal node. In the absence of lineage-specific expression divergence, branch lengths are all approximately equal, regardless of whether the gene expression profile is (*A*) conserved or (*B*) diverged among the species. In contrast, (*C*) lineage-specific expression divergence results in one disproportionally long branch on the lineage in which it occurred (leading to species *X*).

The main objective of this study was to characterize lineage-specific expression divergence in *B. distachyon*, *O. sativa japonica*, and *S. bicolor*. To accomplish this goal, I designed a summary statistic that quantifies lineage-specific expression divergence of genes in three species. In particular, I considered an unrooted tree of three orthologous genes, in which each branch length represents the amount of expression divergence that occurred along a particular lineage (fig. 1). In the absence of lineage-specific expression divergence, all branches lengths should be approximately equal, regardless of whether the gene expression profile is relatively conserved (fig. 1*A*) or diverged (fig. 1*B*) among the species. In contrast, lineage-specific expression divergence should result in a tree with one disproportionally long branch (fig. 1*C*). Thus, my estimate of lineage-specific expression divergence, LED, computes branch lengths on such a tree via application of equation 11.20 in Felsenstein (2004) to gene expression profiles. For example, lineage-specific expression divergence of a gene in species *X* (e.g., Figure 1) can be estimated as ${\mathrm{LED}}_{X}=\frac{1}{2}\left(E_{X,Y}+E_{X,Z}-E_{Y,Z}\right)$, where $E_{X,Y}$, $E_{X,Z}$, and$E_{Y,Z}$ represent pairwise gene expression divergences between species.

For my analysis, I used Euclidian distances between expression profiles to quantify pairwise gene expression divergences between species ($E_{X,Y}$, $E_{X,Z}$, and$E_{Y,Z}$; see Materials and Methods for details). The main advantages of Euclidian distance over alternative distance- and correlation-based metrics are that it is robust to measurement error, and that its squared value increases linearly with evolutionary time (Pereira et al. 2009), such that the Euclidian distance between expression profiles of orthologous genes is expected to increase with evolutionary divergence between species. Yet, regardless of the amount of evolutionary divergence between species, the tendency toward conservation of expression profiles between orthologs yields a right-skewed distribution of Euclidian distances (Pereira et al. 2009; Assis and Bachtrog 2013). Therefore, the mass of a distribution reflects the genome-wide level of expression divergence between a pair of species and is proportional to their evolutionary divergence, and the position of a particular gene within a distribution is indicative of its expression divergence relative to other genes in the genome. Similarly, because LED estimates Euclidian distance along a particular lineage of a three-taxon unrooted tree, the mass of its distribution represents the genome-wide level of expression divergence that occurred along that lineage and is proportional to its evolutionary divergence from the internal node of the tree, whereas the position of a particular gene within the distribution represents its lineage-specific expression divergence relative to other genes in the genome.

**Fig. 2.**
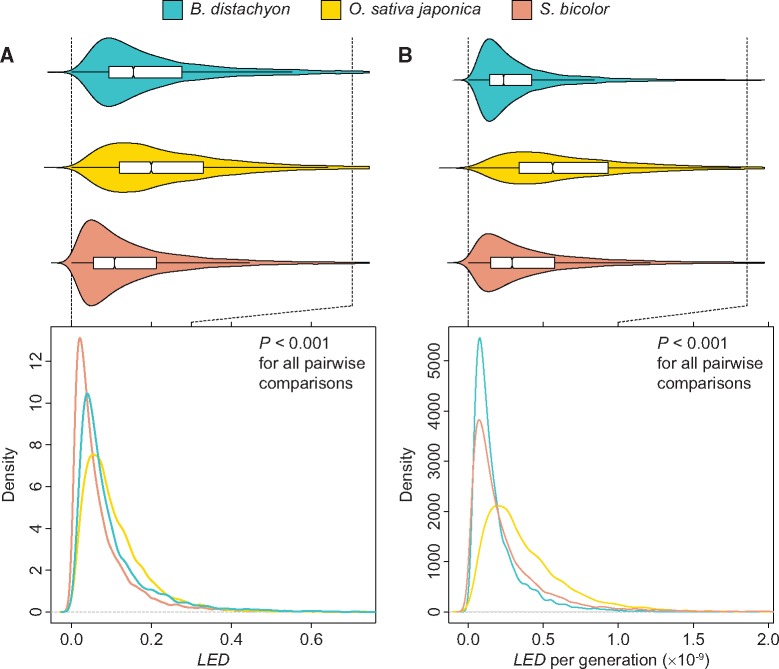
—Comparison of distributions of *LED* among grasses. Notched boxplots embedded in violin plots for (*A*) *LED* and (*B*) *LED* per generation in *B. distachyon*, *O. sativa japonica*, and *S. bicolor*.

To assess lineage-specific expression divergence in grasses, I computed LED for all 1:1:1 orthologous genes in *B. distachyon*, *O. sativa japonica*, and *S. bicolor* (supplementary table S1, Supplementary Material online), using gene expression profiles constructed from nine tissues in the three species (Kapushesky et al. 2010; Davidson et al. 2012; see Materials and Methods for details). As expected, distributions of LED are right-skewed in all species (fig. 2*A*). Moreover, distributions differ significantly among species, such that LED is generally smallest in *S. bicolor*, intermediate in *B. distachyon*, and largest in *O. sativa japonica* (P<0.001for all pairwise comparisons, two-sample permutation tests; see Materials and Methods for details). However, these differences do not account for evolutionary time separating the three species. Therefore, I scaled the distribution of LED for each species by the total number of generations of evolution along its branch. Because generation times vary across climates and growing conditions, I obtained the number of days to anthesis (35 in *B. distachyon*, 65 in *O. sativa japonica*, and 75 in *S. bicolor*) from the study in which RNA-seq data used to compute LED were collected (Davidson et al. 2012), which are comparable to those estimated from other studies (Brkljacic et al. 2011). Then I estimated the total number of generations along each branch by multiplying the number of generations per year in the respective species (365/35 in *B. distachyon*, 365/65 in *O. sativa japonica*, and 365/75 in *S. bicolor*) by the millions of years of evolution from the central node of the three-taxon tree (50 for *B. distachyon*, 50 for *O. sativa japonica*, and 60 for *S. bicolor;* Paterson et al. 2009; Reineke et al. 2011). As with LED (fig. 2*A*), distributions of LED per generation differ significantly among species (fig. 2*B*, P<0.001for all pairwise comparisons, two-sample permutation tests; see Materials and Methods for details), though the ordering of species is altered. In particular, when accounting for evolutionary time from the internal node of the tree, LED is generally smallest in *B. distachyon*, intermediate in *S. bicolor*¸ and largest in *O. sativa japonica*. What is interesting about this finding is that one might expect *B. distachyon* to experience the fastest rate of lineage-specific expression divergence due to increased mutation rates from a shorter generation time (Reineke et al. 2011) and increased efficiency of natural selection from a larger effective population size (Ai et al. 2012; Adugna 2014; Stritt et al. 2018). Yet, *B. distachyon* is also the only species considered whose evolutionary history has not been impacted by domestication. Further, it is intriguing that LED is largest in *O. sativa japonica*, as recent estimates indicate that *O. sativa japonica* underwent domestication ∼4,000 years earlier than *S. bicolor* (Winchell et al. 2017; Zuo et al. 2017). Therefore, these differences support the hypothesis that domestication may have increased the rate of lineage-specific expression divergence in grasses.

### Relationships between LED and Genic Properties

Gene expression divergence has been associated with protein-coding sequence evolution, expression breadth, and network connectivity in a number of diverse species (Makova and Li 2003; Meiklejohn et al. 2003; Nuzhdin et al. 2004; Jordan et al. 2005; Lemos et al. 2005; Duret and Mouchiroud 2000; Ge et al. 2001; Pal et al. 2001; Herbeck et al. 2003; Rocha and Danchin 2004; Subramanian and Kumar 2004; Bhardwaj and Lu 2005; Baerenfaller et al. 2008; French and Pavlidis 2011; Assis 2014; Assis and Kondrashov 2014; Sartor et al. 2006; Assis et al. 2012; Assis and Bachtrog 2013, 2015; Hunt et al. 2013; Gossmann et al. 2016; Hodgins et al. 2016; Mähler et al. 2017). In particular, expression divergence is often positively, though imperfectly, correlated with nonsynonymous sequence divergence (Makova and Li 2003; Nuzhdin et al. 2004; Jordan et al. 2005; Pal et al. 2001; Rocha and Danchin 2004; Subramanian and Kumar 2004; Assis and Kondrashov 2014; Lemos et al. 2005; Sartor et al. 2006; Assis et al. 2012; Assis and Bachtrog 2013, 2015; Hunt et al. 2013; Gossmann et al. 2016; Hodgins et al. 2016; Mähler et al. 2017), suggesting that changes in both encoded proteins and their regulatory sequences contribute to expression divergence. Additionally, previous studies have uncovered strong positive correlations between expression divergence and tissue specificity of genes (Meiklejohn et al. 2003; Jordan et al. 2005; Assis et al. 2012; Duret and Mouchiroud 2000; Assis 2014; Assis and Bachtrog 2013, 2015; Gossmann et al. 2016), indicating that expression divergence is greatest in genes that are expressed in a single tissue and smallest in broadly expressed housekeeping genes. In plants and animals, genes with the greatest levels of expression divergence are often primarily expressed in male tissues (Meiklejohn et al. 2003; Nuzhdin et al. 2004; Assis et al. 2012; Assis 2014; Assis and Bachtrog 2013, 2015; Gossmann et al. 2016), which may be a result of faster male-biased evolution. Last, genes with increased expression divergence are typically located at the edges of gene interaction networks (Lemos et al. 2005; Assis and Bachtrog 2013; Mähler et al. 2017), perhaps because changes in such genes impact fewer pathways and are therefore more likely to be tolerated and retained. Hence, because LED is an estimate of expression divergence, I hypothesized that it would be similarly associated with these genic properties.

**Fig. 3.**
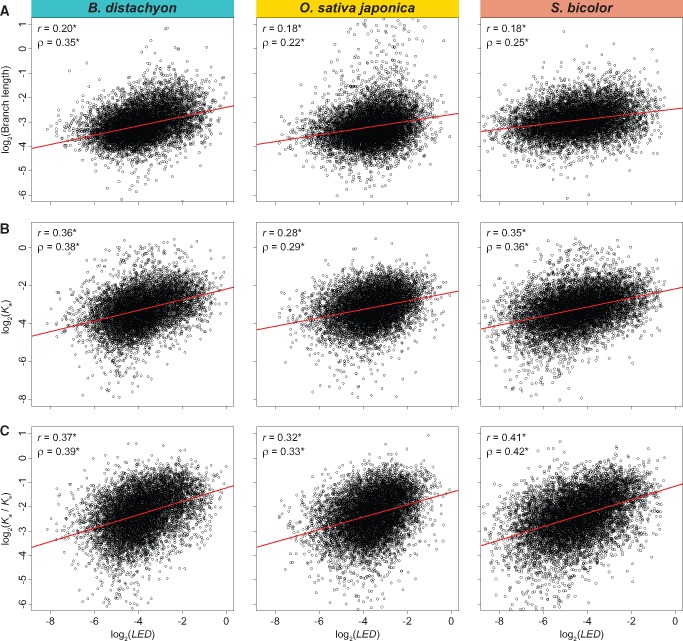
—Relationships between *LED* and protein-coding sequence divergence in grasses. Scatterplots showing correlations between *LED* and (*A*) gene tree branch length, (*B*) nonsynonymous sequence divergence ($K_{a}$), and (*C*) nonsynonymous/synonymous sequence divergence rates ($K_{a}/K_{s}$) in *B. distachyon* (left), *O. sativa japonica* (middle), and *S. bicolor* (right). The best-fit linear regression line is shown in red, and Pearson’s (r) and Spearman’s (ρ) correlation coefficients are provided at the bottom right of each panel. *P<0.001 (see Materials and Methods for details).

To examine the relationship between LED and protein-coding sequence evolution, I computed Pearson’s (r) and Spearman’s (ρ) correlation coefficients between LED and gene tree branch length, nonsynonymous sequence divergence ($K_{a}$), and nonsynonymous/synonymous sequence divergence rates ($K_{a}/K_{s}$; see Materials and Methods for details). As expected, LED is positively correlated with all three measures of protein-coding sequence divergence (fig. 3). However, similar to findings in other species (Makova and Li 2003; Nuzhdin et al. 2004; Pal et al. 2001; Rocha and Danchin 2004; Subramanian and Kumar 2004; Assis and Kondrashov 2014; Jordan et al. 2005; Lemos et al. 2005; Sartor et al. 2006; Assis et al. 2012; Assis and Bachtrog 2013, 2015; Hunt et al. 2013; Gossmann et al. 2016; Hodgins et al. 2016; Mähler et al. 2017), correlations are moderate. Therefore, although lineage-specific expression divergence is associated with changes in protein-coding sequences, it is likely that this relationship is due to similar selective forces acting on coding and regulatory regions of genes. Thus, this result further highlights the role of regulatory variation in gene expression and phenotypic evolution.

**Fig. 4.**
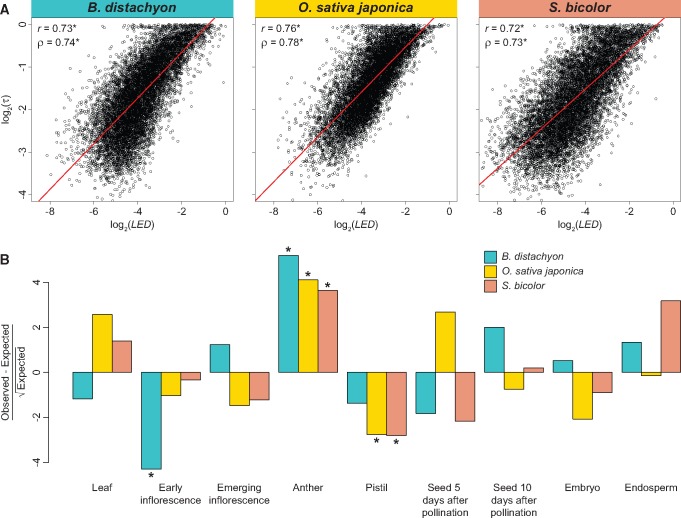
—Relationships between LED and tissue-specific gene expression in grasses. (*A*) Scatterplots showing correlations between LED and tissue specificity (τ) in *B. distachyon* (left), *O. sativa japonica* (middle), and *S. bicolor* (right). The best-fit linear regression line is shown in red, and Pearson’s (r) and Spearman’s (ρ) correlation coefficients are provided at the bottom right of each panel. (*B*) Hanging Chi-gram depicting differences between observed counts of primary tissues in genes with top 1% LED relative to those expected based on proportions of primary tissues of all genes. Positive values indicate overrepresentations, and negative values underrepresentations, relative to expectations. *P<0.001 (see Materials and Methods for details).

Next, I investigated the association between LED and expression breadth by calculating Pearson’s (r) and Spearman’s (ρ) correlation coefficients between LED and the tissue specificity index τ (Yanai et al. 2005). As expected, LED is significantly and strongly positively correlated with τ (fig. 4*A*), indicating that increased lineage-specific expression divergence is primarily due to tissue-specific changes. To further examine this phenomenon, I selected genes in the top 1% of LED and classified each gene by the tissue in which it is primarily expressed. Comparisons of primary tissues of these genes with large LED to those expected based on genome-wide counts (see Materials and Methods for details) revealed significant overrepresentations of anther expression in all three species (fig. 4*B*). Because anther is the organ that produces pollen in plants, its overrepresented expression in genes with large LED suggests that lineage-specific expression divergence is often associated with male-biased evolution, as has been found for expression divergence in many plant and animal species (Meiklejohn et al. 2003; Nuzhdin et al. 2004; Assis et al. 2012; Assis 2014; Assis and Bachtrog 2013, 2015; Gossmann et al. 2016). Moreover, there is a significant underrepresentation of early inflorescence expression among genes with large LED in *B. distachyon*, and in pistil expression among genes with large LED in both *O. sativa japonica* and *S. bicolor*. Thus, underrepresented tissues of genes with large LED differ between wild and domesticated species, perhaps pointing to a role of domestication in tissue targets of lineage-specific expression divergence.

Last, I assessed the relationship between LED and network connectivity in grasses. To estimate the network connectivity of each gene, I obtained the number of its known interaction partners from experimental studies (see Materials and Methods for details). Because count data are not continuous, I was unable to estimate correlation coefficients between LED and network connectivity. Rather, I performed Poisson regression on these data in each species (see Materials and Methods for details), yielding regression coefficients β=-1.27 for *B. distachyon*, β=-0.93 for *O. sativa japonica*, and β=-1.02 for *S. bicolor* ($P<2.0\times 10^{-16}$ for all regressions). Hence, consistent with findings for expression divergence between species (Lemos et al. 2005; Assis and Bachtrog 2013; Ge et al. 2001; Bhardwaj and Lu 2005; French and Pavlidis 2011; Assis and Kondrashov 2014; Mähler et al. 2017), there is a significant negative relationship between LED and network connectivity, such that lineage-specific expression divergence often targets lowly connected genes at the edges of interaction networks.

### Relationship between LED and Gene Function 

Though protein-coding sequence evolution, expression breadth, and network connectivity can each shed light on different aspects of gene function, none of these metrics provides a complete picture of the role of a gene within a biological system. Therefore, to better understand the functional modules targeted by lineage-specific expression divergence in grasses, I utilized annotation data from the Gene Ontology (GO) Consortium (Ashburner et al. 2000; Gene Ontology Consortium 2017). In particular, GO terms classify genes by the molecular functions that they perform, the cellular components in which they perform these functions, and the larger-scale biological processes in which they participate (Ashburner et al. 2000; Gene Ontology Consortium 2017). To study the relationship between LED and GO terms in each species, I sorted genes by their LED, performed GO enrichment analysis on ranked lists, and extracted significantly overrepresented GO terms (supplementary tables S2–S4, Supplementary Material online; see Materials and Methods for details).

Consistent with anther-biased expression patterns, I observed enrichment of GO terms related to anther function in *O. sativa japonica* and *S. bicolor*. In particular, the biological process “microsporogenesis” is enriched in both species, and the biological process “recognition of pollen” is enriched in *S. bicolor*. Microsporogenesis is the production of microspores that give rise to pollen (Ashburner et al. 2000; Gene Ontology Consortium 2017), thus implicating lineage-specific expression divergence in pollen development in *O. sativa japonica* and *S. bicolor*. In contrast, recognition of pollen is crucial in self-incompatibility, a strategy used by plants to limit or prevent self-fertilization (Ashburner et al. 2000; Gene Ontology Consortium 2017). Because self-fertilization reduces genetic variation and has been described as an evolutionary dead end (Stebbins 1957), the enrichment of this term suggests that lineage-specific expression divergence may be associated with increased adaptive potential in *S. bicolor*. Therefore, lineage-specific expression divergence may impact both the development and evolution of pollen in *O. sativa japonica* and *S. bicolor*.

Additionally, I sought to address whether there are any global patterns in functional targets of lineage-specific divergence across grasses. To answer this question, I compared enriched GO terms in *B. distachyon*, *O. sativa japonica*, and *S. bicolor*. Only three terms, one from each GO class, were enriched in all three species: the molecular function “serine-type endopeptidase inhibitor activity,” the cellular component “cell wall,” and the biological process “DNA metabolic process.” Though DNA metabolism is a general process, the other two GO terms point to a role of lineage-specific expression divergence in host-pathogen defense. In particular, plant serine endopeptidase inhibitors, also called serine protease inhibitors, compose a diverse group of genes that are expressed in response to injury or attack by insects or pathogens (Hartl et al. 2011; Jamal et al. 2013). Though their precise mechanisms of action remain to be elucidated, their primary mode of defense is via inhibition of the protease family of digestive enzymes (Hartl et al. 2011; Jamal et al. 2013). Moreover, because the cell wall forms a physical barrier and interface between adjacent cells, it plays a pivotal role in plant-pathogen interactions (Keegstra 2010). Last, it is interesting to note that “defense response” is the most significantly enriched biological process in *O. sativa japonica*. Thus, lineage-specific expression divergence may be primarily associated with host-pathogen defense responses in grasses.

As a complementary approach to understand functional modules impacted by lineage-specific expression divergence, I performed database and literature searches for genes with the top LED in each species: Bradi1g62070 in *B. distachyon*, Os02g0725700 in *O. sativa japonica*, and Sb08g003710 in *S. bicolor*. Bradi1g62070, or LOC100824152, is an uncharacterized protein-coding gene that is part of a family of only three genes, one in each of the species considered in this study. Therefore, this gene family is likely young. Because the annotation of Bradi1g62070 is limited, I investigated its orthologs OS03G0356540 in *O. sativa japonica* and SB07G003950 in *S. bicolor*. Unfortunately, information about SB07G003950 is also scarce. However, OS03G0356540, better known as *CXXC1*, is a small cysteine-rich protein-coding gene with CXXC and CXXXC motifs that performs functions in a cytoplasmic membrane-bounded vesicle (GO:0016023; Ashburner et al. 2000; Gene Ontology Consortium 2017). Though this particular gene has not been closely examined, small cysteine-rich genes often possess a diversity of lineage-specific antimicrobial roles in plants (Silverstein et al. 2007). Therefore, lineage-specific expression divergence of Bradi1g62070 may be related to defense against a microbial pathogen that specifically targeted the *B. distachyon* lineage.

Os02g0725700, better known as *OsHAP3E*, is a member of the Heterotrimeric Heme Activator (HAP) family of genes. It is a histone-fold domain containing gene that is involved in vegetative and reproductive development (Ito et al. 2011; Zhang and Xue 2013). A recent study of 35 HAP genes in rice identified *OsHAP3E* as one of just four genes whose overexpression delayed flowering time under long-day conditions (Li et al. 2016). Moreover, of these four genes, *OsHAP3E* had the largest and most significant effect on flowering time (Li et al. 2016). Flowering time is a crucial determinant of rice domestication, as it regulates seasonal and geographic adaptability (Izawa 2007). Further, delayed flowering time increases yield (Xue et al. 2008; Wei et al. 2010), an important phenotype in domestication of crops for human consumption. Therefore, lineage-specific expression divergence of *OsHAP3E* may be associated with domestication pressures in *O. sativa japonica*.

Sb08g003710, also known as *NHL3*, produces a protein with an immunoglobulin-like fold that is involved in host-pathogen defense in *S. bicolor* (Upadhyaya et al. 2013). In particular, a recent study demonstrated that *NHL3* is one of a handful of genes associated with resistance to anthracnose, a fungal disease considered to be one of the most common and destructive in *S. bicolor* (Upadhyaya et al. 2013). Moreover, *NHL3* is homologous to the gene *HIN1*, which has been shown to mediate the hypersensitive response in tobacco and tomato plants (Gopalan et al. 1996). Hypersensitive response is a major defense mechanism to anthracnose in *S. bicolor* that causes the rapid death of plant cells at the infection site. Thus, defense against this widespread fungal disease may have contributed to the dramatic lineage-specific expression divergence of *NHL3* in *S. bicolor*.

## Discussion

In this article, I present the first genome-wide analysis of lineage-specific expression divergence in the grasses *B. distachyon*, *O. sativa japonica*, and *S. bicolor*. To estimate lineage-specific expression divergence in these species, I design a novel branch-based summary statistic, LED. Contrary to this approach, past studies in other taxa have primarily utilized model-based estimates of lineage-specific expression divergence (Cáceres et al. 2003; Gu and Gu 2003; Rifkin et al. 2003; Gu 2004; Khaitovich et al. 2005; Gilad et al. 2006; Blekhman et al. 2008; Chaix et al. 2008; Brawand et al. 2011; Kayserili et al. 2012; Perry et al. 2012; Rohlfs and Nielsen 2015). In particular, a recent focus of such work has been in implementing Ornstein–Uhlenbeck (OU) processes, which can model evolution of gene expression levels along phylogenetic trees (Hansen 1997; Butler and King 2004; Bedford and Hartl 2009; Brawand et al. 2011; Perry et al. 2012; Rohlfs et al. 2014; Rohlfs and Nielsen 2015). Because OU processes model Brownian motion with a pull toward an optimal state, they have a natural application to evolution, in which drift is analogous to Brownian motion, selection to pull, and fittest phenotype to optimal state (Hansen 1997; Butler and King 2004). Therefore, OU processes have high power for detecting shifts in gene expression due to lineage-specific expression divergence. However, the major advantages of LED over these approaches are its lack of assumptions and inherent simplicity. Specifically, OU processes assume normality and require information about tree topology and divergence times. Moreover, when data comprise multiple tissues as in the current analysis, further assumptions are made about the covariance structure among tissues (e.g., independence, equal variance, etc.). In contrast, the only information necessary for calculating LED is the genome-wide expression levels from at least one tissue, developmental stage, or experimental condition in three related species. Further, as with OU processes, LED can be applied to any measurable quantitative trait, enabling the assessment of lineage-specific divergence of a wide array of genetic, epigenetic, and phenotypic attributes.

A limitation of LED is that it is applied to three-species trees. In particular, consider figure 1*C*, which illustrates disproportionally large LED along the lineage leading to ortholog *X*. Rather than a change arising along the lineage leading to *X*, it is also possible that a change occurred along the lineage ancestral to *Y* and *Z*. Rooting the tree would not resolve this issue because it would require assigning the outgroup state as ancestral, thereby incorporating an additional assumption and removing the outgroup species from the analysis. Rather, an optimal solution is to obtain similar data from a fourth species. Then, one can construct an analogous statistic to LED that subtracts out the internal branch length of the four-species tree via application of equation 12.6 in Felsenstein (2004). Unfortunately, similar RNA-seq data do not currently exist for a fourth grass species, and so this approach is not feasible in the present study. However, even with this three-species approach, it is unlikely that changes ancestral to two of the species are common phenomena in the grasses studied here. In particular, the findings that LED is positively correlated with protein-coding sequence divergence and tissue specificity, negatively correlated with network connectivity, and positively associated with high male tissue expression and GO terms related to male reproduction and host-pathogen defense are all consistent with those from previous studies of gene expression divergence in many plants and animals (Makova and Li 2003; Meiklejohn et al. 2003; Nuzhdin et al. 2004; Jordan et al. 2005; Lemos et al. 2005; Sartor et al. 2006; Silverstein et al. 2007; Assis 2014; Assis et al. 2012; Assis and Bachtrog 2013, 2015; Hunt et al. 2013; Gossmann et al. 2016; Hodgins et al. 2016; Mähler et al. 2017). Therefore, LED is likely capturing general patterns of lineage-specific expression divergence in grasses.

Comparison of distributions of LED among grasses revealed that lineage-specific expression divergence occurs at the slowest rate in the wild *B. distachyon*. This result is unexpected given increased mutation rates due to shorter generation time (Reineke et al. 2011) and decreased efficiency of natural selection due to larger effective population size (Ai et al. 2012; Adugna 2014; Stritt et al. 2018) of *B. distachyon* relative to *O. sativa japonica* and *S. bicolor*. It is also intriguing that LED occurs at the fastest rate in *O. sativa japonica* because it was domesticated several thousand years earlier than *S. bicolor* (Winchell et al. 2017; Zuo et al. 2017). Thus, rates of lineage-specific expression divergence support the hypothesis that lineage-specific expression divergence in grasses may be influenced by their domestication histories. However, of key importance is that these grass species also differ in their genomic contents, climate, environmental conditions, and pathogens. Moreover, *O. sativa japonica* and *S. bicolor* were domesticated in different locations of the world and may have each experienced multiple rounds of domestication that selected for varied traits (Doebley et al. 2006; Glémin and Bataillon 2009; Winchell et al. 2017). Therefore, though differences in LED are consistent with domestication and its timing, there are many variables—both related and unelated to domestication—that may affect lineage-specific expression divergence of grasses.

In all grasses, increased LED is associated with increased protein-coding sequence divergence, increased tissue specificity, and decreased network connectivity. These relationships are not unexpected given similar findings for expression divergence between species in other taxa (Makova and Li 2003; Meiklejohn et al. 2003; Nuzhdin et al. 2004; Jordan et al. 2005; Lemos et al. 2005; Duret and Mouchiroud 2000; Ge et al. 2001; Pal et al. 2001; Herbeck et al. 2003; Rocha and Danchin 2004; Subramanian and Kumar 2004; Bhardwaj and Lu 2005; Baerenfaller et al. 2008; French and Pavlidis 2011; Assis 2014; Assis and Kondrashov 2014; Sartor et al. 2006; Assis et al. 2012; Assis and Bachtrog 2013, 2015; Hunt et al. 2013; Gossmann et al. 2016; Hodgins et al. 2016; Mähler et al. 2017). Therefore, it appears that expression divergence in general affects nonhousekeeping genes, perhaps in which it is more likely to be tolerated. Moreover, examination of the primary tissues in which genes with large LED are expressed uncovered strong biases toward anther expression in all three species, consistent with the faster sequence and expression evolution of male-biased genes observed in both plants and animals (Meiklejohn et al. 2003; Assis 2014; Nuzhdin et al. 2004; Assis et al. 2012; Assis and Bachtrog 2013, 2015; Gossmann et al. 2016). Hypotheses for faster evolution of male-biased genes include increased mutation rates due to a greater number of germline cell divisions in male tissues (Shimmin et al. 1993), positive selection due to sexual selection (Pröschel et al. 2006; Ellegren and Parsch 2007), and relaxed negative selection due to reduced functional pleiotropy (Ellegren and Parsch 2007; Gershoni and Pietrokovski 2014; Harrison et al. 2015). In dioecious plants, there are more cell divisions during pollen than ovule production, and male-biased genes often have higher mutation rates (Filatov and Charlesworth 2002; Whittle and Johnston 2002), both of which support the increased mutation rate hypothesis (Shimmin et al. 1993). However, the positive association between LED and protein-coding sequence divergence is indicative of positive selection, whereas the positive association between LED and tissue specificity points to decreased pleiotropy and relaxed negative selection. Therefore, any of these mechanisms may contribute to increased male-biased evolutionary rates in grasses.

Functional analyses uncovered three major biological themes associated with lineage-specific expression divergence in grasses: male reproduction, host-pathogen defense, and domestication. Male reproduction is not a surprising result in light of the male-biased expression evolution observed. However, host-pathogen defense is noteworthy because its associated GO terms are enriched in all three species, “pathogen defense” is the most significantly enriched GO biological process in *O. sativa japonica*, and genes with the largest LED in both *B. distachyon* and *S. bicolor* are likely involved in defense against pathogens. Therefore, host-pathogen defense appears to be a major functional target of lineage-specific expression divergence in all grasses. Further, it is interesting to note that male reproduction and host-pathogen defense may be associated with one another, in that infection with pathogens has been found to alter male-biased expression (Zemp et al. 2015), sexual dimorphic traits (Zemp et al. 2015), and DNA methylation patterns (Castellano et al. 2016) in other dioecious plants. In contrast to the other two functions, there do not appear to be any enriched GO terms specifically related to domestication. Yet comparison of overall rates of LED among the three species suggests that increased lineage-specific expression divergence may be associated with domestication, and the gene with the largest LED in *O. sativa japonica* may play an important role in enhanced adaptation and yield during domestication. Therefore, these findings indicate that lineage-specific expression divergence in grasses may increase under domestication and target rapidly evolving genes involved in male reproduction, host-pathogen defense, and phenotypes selected for during domestication.

It is important to note that conclusions relating lineage-specific expression divergence to domestication are limited in this study. In particular, phenotypic evolution under domestication of grasses occurred over short evolutionary timescales (Glémin and Bataillon 2009) that were not examined in the current analysis. Therefore, signals of lineage-specific expression divergence associated with domestication are likely intermingled with those due to unrelated events preceding or following domestication. As a result, though the increased rate of lineage-specific expression divergence in domesticated grasses is consistent with faster evolutionary divergence under domestication, changes in specific genes or functions cannot be directly attributed to domestication. Similarly, lineage-specific expression divergence of genes involved in male reproduction or host-pathogen defense may or may not be associated with domestication. Indeed, such genes undergo rapid evolution in many species of undomesticated plants and animals (e.g., Meiklejohn et al. 2003; Nuzhdin et al. 2004; Silverstein et al. 2007; Assis et al. 2012; Assis 2014; Assis and Kondrashov 2014; Assis and Bachtrog 2013, 2015; Gossmann et al. 2016). Therefore, it is likely that lineage-specific expression divergence affects a suite of gene functions that are targeted by selection pressures independently related to male reproduction, host-pathogen defense, and domestication of grasses.

## Materials and Methods

### Identification of Orthologous Genes

Amino acid sequences of protein-coding genes in *B. distachyon* (version 1.0; International Brachypodium Initiative 2010), *O. sativa japonica* (IRGSP-1.0; International Rice Genome Sequencing Project 2005), and *S. bicolor* (version 1.4; Patterson et al. 2009) were downloaded from EnsemblPlants (release 37) at https://plants.ensembl.org. Orthologous groups were identified by running OrthoMCL (version 2.0; Li et al. 2003; Chen et al. 2006; Fischer et al. 2011) with default parameters on the longest transcripts of all genes in the three species. In total, 29,970 orthologous groups were identified. However, to minimize the probability of misassigning orthologs due to duplication, I limited my analysis to 11,142 1:1:1 orthologs.

### Gene Expression Analyses

Tables of normalized RNA-seq abundances in transcripts per million (TPM) for nine tissues of *B. distachyon*, *O. sativa japonica*, and *S. bicolor* were downloaded from Expression Atlas (Kapushesky et al. 2010) at https://www.ebi.ac.uk/gxa/home/; last accessed December 12, 2018. These data were obtained with the iRAP pipeline, averaged across technical replicates, and quantile normalized (Papatheodorou et al. 2018). The tissues included in these tables are leaf, early inflorescence, emerging inflorescence, anther, pistil, seed 5 days after pollination, seed 10 days after pollination, embryo, and endosperm (Davidson et al. 2012). Data were log-transformed, genes with log_2_(TPM) >2 in at least one of the nine tissues were retained, and expression profiles were constructed from relative abundance levels to enable cross-species comparisons (Liao and Zhang 2006; Pereira et al. 2009). Euclidian distances were calculated between expression profiles in all nine tissues of orthologous genes for each pair of species ($E_{B,O}$, $E_{B,S}$, and$E_{O,S}$). For example, $E_{B,O}=\sqrt{\sum_{t=1}^{9}{\left(B_{t}-O_{t}\right)}^{2}}$, where t represents one of the nine tissues, $B_{t}$ the relative expression of the *B. distachyon* ortholog in tissue t, and $O_{t}$ the relative expression of the *O. sativa japonica* ortholog in tissue t. LED was computed from $E_{B,O}$, $E_{B,S}$, and$E_{O,S}$. The tissue specificity index τ, which ranges from 0 (broadly expressed) to 1 (tissue-specific), was used to estimate expression breadth of genes (Yanai et al. 2005).

### Sequence Analyses

Protein-coding sequences of genes in *B. distachyon* (version 1.0; International Brachypodium Initiative 2010), *O. sativa japonica* (IRGSP-1.0; International Rice Genome Sequencing Project 2005), and *S. bicolor* (version 1.4; Patterson et al. 2009) were downloaded from EnsemblPlants (release 37) at https://plants.ensembl.org; last accessed December 12, 2018. Multiple alignments of 1:1:1 orthologs were performed with MACSE (version 1.2; Ranwez et al. 2011), which accounts for frameshifts and stop codons. PhyML (version 3.0; Guindon et al. 2010) was used to calculate branch lengths for each gene tree, and the codeml function in PAML (version 4; Yang 2007) was used to calculate $K_{a}$ and $K_{a}/K_{s}$ for all pairs of orthologs.

### Network Connectivity Analyses

Gene interaction data for *B. distachyon*, *O. sativa japonica*, and *S. bicolor* were downloaded from the STRING database at https://string-db.org; last accessed December 12, 2018 (Snel et al. 2000; Szklarczyk et al. 2017). The number of interaction partners of each gene was estimated as the total number of unique genes with which an interaction was recorded from experimental data. The relationship between LED and number of interaction partners was assessed with Poisson regression. In particular, a Poisson model was fit to the data for each species and used to estimate regression coefficients and compute their P-values. Fits of Poisson models to the data were ensured by performing $X^{2}$ goodness-of-fit tests on residual deviance (P=1 for tests in all species).

### GO Analyses

GO annotation data sets for *B. distachyon*, *O. sativa japonica*, and *S. bicolor* were downloaded from the PLAZA 4.0 database at https://bioinformatics.psb.ugent.be/plaza/; last accessed December 12, 2018 (Van Bel et al. 2018). TopGo (Alexa et al. 2006) was used to assess GO enrichment of genes in each species based on their ranked LED scores. For each analysis, the Kolmogorov–Smirnov test was applied, using the weight01 algorithm to account for the GO topology. GO terms with fewer than ten annotated genes were not considered in analyses (by setting nodeSize = 10), and only those with P<0.01 were classified as significantly enriched (supplementary tables S1–S3, Supplementary Material online).

### Statistical Analyses

All statistical analyses were performed in the R software environment (R Core Team 2013). Two-sample permutation tests were used to evaluate differences between pairs of distributions shown in figure 2. Each test was performed with 1,000 permutations and with the test statistic set as the difference between medians. Correlations depicted in figures 3 and 4*A* were assessed with one-sample t-tests. For results shown in figure 4*B*, $X^{2}$goodness-of-fit tests were first used to compare observed distributions of highest-expressed tissues in genes with the top 1% of LED scores with their expected distributions based on highest-expressed tissues in all genes in the genome. Because observed distributions were significantly different from those expected in all species (P<0.001), binomial tests were performed to compare the observed frequency of each primary tissue class in genes with the top 1% of LED with the genome-wide frequency of the class. In each test, the number of successes X was set as the count for a particular primary tissue class, the number of trials n as the total number of genes with the top 1% of LED in each species, and the probability of success pas the frequency of the primary tissue class in the genome of the species of interest. P-values from binomial tests were Bonferroni-adjusted to correct for the nine comparisons performed.

## Supplementary Material

Supplementary data are available at *Genome Biology and Evolution* online.

## Supplementary Material

Supplementary Data
