# Supplementary material for: Lineage-Specific Expression Divergence in Grasses Is Associated with Male Reproduction, Host-Pathogen Defense, and Domestication
Source: Genome Biol Evol. 2018 Nov 6;11(1):207–19. doi: 10.1093/gbe/evy245 (PMC6331041; doi:10.1093/gbe/evy245)
Supplement: Supplementary Data [file evy245_supp.zip › Assis_GBE2018_SupportingInformationLegends.pdf]

SUPPORTING INFORMATION LEGENDS

Table S1. *LED* of 1:1:1 orthologs in *B. distachyon*, *O. sativa japonica*, and *S. bicolor*

Table S2. Enriched GO terms in *B. distachyon*

Table S3. Enriched GO terms in *O. sativa japonica*

Table S4. Enriched GO terms in *S. bicolor*
