# Supplementary material for: Lineage-Specific Expression Divergence in Grasses Is Associated with Male Reproduction, Host-Pathogen Defense, and Domestication
Source: Genome Biol Evol. 2018 Nov 6;11(1):207–19. doi: 10.1093/gbe/evy245 (PMC6331041; doi:10.1093/gbe/evy245)
Supplement: Supplementary Data [file evy245_supp.zip › Assis_GBE2018_TablesS2-S4.pdf]

Table S2. Enriched GO terms in *B. distachyon*

Class	ID	Term	P
MF	GO:0016758	transferase activity, transferring hexosyl groups	0.00022
	GO:0045735	nutrient reservoir activity	0.00026
	GO:0008234	cysteine-type peptidase activity	0.00058
	GO:0004867	serine-type endopeptidase inhibitor activity	0.00150
	GO:0008146	sulfotransferase activity	0.00151
	GO:0042626	ATPase activity, coupLED to transmembrane movement of substances	0.00161
	GO:0016788	hydrolase activity, acting on ester bonds	0.00215
	GO:0016760	cellulose synthase (UDP-forming) activity	0.00309
	GO:0004713	protein tyrosine kinase activity	0.00327
	GO:0003677	DNA binding	0.00344
	GO:0004185	serine-type carboxypeptidase activity	0.00828
	GO:0004499	N,N-dimethylaniline monooxygenase activity	0.00980
	GO:0019843	rRNA binding	0.00983
CC	GO:0016021	integral component of membrane	0.00017
	GO:0015935	small ribosomal subunit	0.00038
	GO:0009539	photosystem II reaction center	0.00047
	GO:0000312	plastid small ribosomal subunit	0.00048
	GO:0005618	cell wall	0.00071
	GO:0005730	nucleolus	0.00193
	GO:0000148	1,3-beta-D-glucan synthase complex	0.00348
	GO:0005615	extracellular space	0.00358
	GO:0009523	photosystem II	0.00682
BP	GO:0006259	DNA metabolic process	0.00028
	GO:0006260	DNA replication	0.00056
	GO:0006754	ATP biosynthetic process	0.00112
	GO:0019538	protein metabolic process	0.00124
	GO:0006334	nucleosome assembly	0.00206
	GO:0009169	purine ribonucleoside monophosphate catabolic process	0.00280
	GO:0070417	cellular response to cold	0.00408
	GO:0015991	ATP hydrolysis coupLED proton transport	0.00557
	GO:0015977	carbon fixation	0.00668
	GO:0006139	nucleobase-containing compound metabolic process	0.00894
	GO:0009944	polarity specification of adaxial/abaxial axis	0.00954

MF = molecular function, CC = cellular component, BP = biological process

Table S3. Enriched GO terms in *O. sativa japonica*

Class	ID	Term	P
MF	GO:0043531	ADP binding	$< 10^{-30}$
	GO:0032550	purine ribonucleoside binding	$< 10^{-30}$
	GO:0004523	RNA-DNA hybrid ribonuclease activity	0.00018
	GO:0004386	helicase activity	0.00022
	GO:0003735	structural constituent of ribosome	0.00035
	GO:0004650	polygalacturonase activity	0.00191
	GO:0016829	lyase activity	0.00212
	GO:0008146	sulfotransferase activity	0.00244
	GO:0016760	cellulose synthase (UDP-forming) activity	0.00346
	GO:0030598	rRNA N-glycosylase activity	0.00499
	GO:0008289	lipid binding	0.00626
	GO:0031625	ubiquitin protein ligase binding	0.00668
	GO:0004867	serine-type endopeptidase inhibitor activity	0.00728
	GO:0004803	transposase activity	0.00865
CC	GO:0005618	cell wall	0.00040
	GO:0005739	mitochondrion	0.00049
	GO:0000502	proteasome complex	0.00172
	GO:0005840	ribosome	0.00178
	GO:0033180	proton-transporting V-type ATPase, V1 domain F(o)	0.00533
	GO:0031225	anchored component of membrane	0.00549
	GO:0098805	whole membrane	0.00606
BP	GO:0006952	defense response	$< 10^{-30}$
	GO:0006461	protein complex assembly	0.00018
	GO:0006259	DNA metabolic process	0.00106
	GO:0006508	proteolysis	0.00188
	GO:0006412	translation	0.00215
	GO:0046373	L-arabinose metabolic process	0.00418
	GO:0006351	transcription, DNA-templated	0.00448
	GO:0006139	nucleobase-containing compound metabolic process	0.00483
	GO:0006334	nucleosome assembly	0.00488
	GO:0006869	lipid transport	0.00534
	GO:0006915	apoptotic process	0.00545
	GO:0009556	microsporogenesis	0.00701
	GO:0055114	oxidation-reduction process	0.00708
	GO:0006730	one-carbon metabolic process	0.00722
	GO:0016998	cell wall macromolecule catabolic process	0.00896

MF = molecular function, CC = cellular component, BP = biological process

Table S4. Enriched GO terms in *S. bicolor*

Class	ID	Term	P
MF	GO:0008234	cysteine-type peptidase activity	$< 10^{-30}$
	GO:0004386	helicase activity	0.00017
	GO:0016758	transferase activity, transferring hexosyl groups	0.00059
	GO:0003735	structural constituent of ribosome	0.00077
	GO:0010333	terpene synthase activity	0.00082
	GO:0016829	lyase activity	0.00230
	GO:0004867	serine-type endopeptidase inhibitor activity	0.00237
	GO:0045735	nutrient reservoir activity	0.00284
	GO:0005524	ATP binding	0.00381
	GO:0050660	flavin adenine dinucleotide binding	0.00403
	GO:0042626	ATPase activity, coupLED to transmembrane movement of substances	0.00458
	GO:0003677	DNA binding	0.00630
	GO:0008762	UDP-N-acetylmuramate dehydrogenase activity	0.00738
	GO:0003774	motor activity	0.00759
CC	GO:0016021	integral component of membrane	0.00019
	GO:0015935	small ribosomal subunit	0.00020
	GO:0016459	myosin complex	0.00048
	GO:0005618	cell wall	0.00110
	GO:0022627	cytosolic small ribosomal subunit	0.00158
	GO:0009539	photosystem II reaction center	0.00188
	GO:0034399	nuclear periphery	0.00334
	GO:0045263	proton-transporting ATP synthase complex, coupling factor F(o)	0.00355
BP	GO:0006278	RNA-dependent DNA biosynthetic process	$< 10^{-30}$
	GO:0006259	DNA metabolic process	0.00029
	GO:0006412	translation	0.00052
	GO:0048544	recognition of pollen	0.00055
	GO:0006461	protein complex assembly	0.00062
	GO:0009556	microsporogenesis	0.00093
	GO:0006281	DNA repair	0.00110
	GO:0006468	protein phosphorylation	0.00141
	GO:0055114	oxidation-reduction process	0.00152
	GO:0055081	anion homeostasis	0.00785

MF = molecular function, CC = cellular component, BP = biological process
